# Development and validation of diagnostic prediction models for central precocious puberty in girls based on machine learning: a multicenter retrospective study

**DOI:** 10.3389/fendo.2026.1857867

**Published:** 2026-06-15

**Authors:** Wenyong Wu, Zhe Su, Haiyan Wei, Yanhong Li, Benlong Zhu, Xin Yuan, Daibin Lei, Yi Wei, Xian Wu, Hanghan Ou, Xinyu Chen, Ziling Zhu, Ruimin Chen

**Affiliations:** 1 Department of Endocrinology, Genetics and Metabolism, Fuzhou First General Hospital Affiliated with Fujian Medical University (Fuzhou Children’s Hospital of Fujian Province), Fuzhou, Fujian, China; 2 Department of Endocrinology, Shenzhen Children’s Hospital, Shenzhen, Guangdong, China; 3 Department of Endocrinology, Genetics and Metabolism, Henan Children’s Hospital (Children’s Hospital Affiliated to Zhengzhou University), Zhengzhou, Henan, China; 4 Department of Pediatrics, The First Affiliated Hospital, Sun Yat-sen University, Guangzhou, Guangdong, China

**Keywords:** central precocious puberty, girl, machine learning, multicenter, prediction model

## Abstract

**Objective:**

We developed novel diagnostic prediction models for central precocious puberty (CPP) diagnosis in girls based on machine learning.

**Methods:**

In this retrospective study, 2148 girls with PP from 4 centers in China were included, and divided into training group (n = 1048), validation group (n = 262), test group 1 (n = 270), test group 2 (n = 278), and test group 3 (n = 290). Diagnostic prediction models were developed based on logistic regression, linear support vector machine (SVM), RandomForest, XGBoost and fully connected neural network. Model discrimination was assessed with C-statistics and accuracy rates. Model calibration was analyzed by calibration curves.

**Results:**

A total of eight independent predictors were screened by Lasso regression, including chronological age, disease course, height SDS, basal luteinizing hormone, bone age (BA) - chronological age (CA), height SDS for bone age, uterine volume, and larger ovarian volume. Among the five established prediction models, the SVM model demonstrated optimal performance, achieving AUC values of 0.850 in internal validation and 0.827 in external validation, accuracy rates of 78.6% in internal validation and 72.1% average in external validations. The model is displayed on the website: https://wuwenyong.shinyapps.io/CPPpredict/.

**Conclusions:**

The SVM prediction model can assist diagnose CPP. Through the internal and multi-center external validations, the model showed good degree of discrimination and calibration.

## Introduction

Precocious puberty (PP) is classically defined by the appearance of sexual secondary characteristics before the age of 8 years in girls and 9 years in boys (1). However, after a national, cross-sectional, community-based health survey was performed, the diagnostic cut-off point of PP girls was adjusted from 8 years to 7.5 years in China (2, 3). Premature activation of the hypothalamic-pituitary-gonadal axis leads to central precocious puberty (CPP), the most common type of PP, which can begets a lower final adult height and may lead to psychological disturbances (4).

The diagnosis of CPP is dependent on the timing of sexual characteristics, symptoms, and laboratory results. Among them, gonadotropin-releasing hormone (GnRH) stimulation test can discriminate of CPP, peripheral PP, and partial PP (5, 6). However, this test is time-consuming, requires multiple blood samplings, may cause adverse reactions, and is costly. Therefore, establishing a diagnostic approach that can identify CPP without the need for a GnRH stimulation test would be a valuable clinical asset.

Prediction models can be used to predict the probability of CPP diagnosis based on baseline features of patients. Pan L, et al. (7, 8) established a machine learning-based model using predictors of breast stages, pubic hair stages, body composition, basal serum LH, FSH, bone age (BA) assessment, and pelvic ultrasonography to predict CPP in girls. In 2021, You J, et al. (9) proposed a CPP risk score model based on Logistics regression, which classified patients into low-, median-, and high-risk groups. Xu Y, et al. (10) developed a dynamic multimodal variational autoencoder for the prediction of CPP patients with 41 features in 2021. These prediction models provided an exploration of identification tools for CPP patients. Notwithstanding these previous models which focused mostly on development and internal validation, they lacked external validation with independent datasets. Consequently, the generalizability of these models remains unproven. Furthermore, the inclusion of dozens to hundreds of predictors to enhance model performance renders clinical implementation unwieldy and impractical. The previous prediction models have not yet entered the clinical application.

In 2021, our research group enrolled 1,107 girls with PP and developed a CPP diagnostic prediction model based on logistic regression (11). Our model incorporated five clinical indicators as prognosticators: disease course (the time from the onset of breast development to the first consultation), breast Tanner stage, basal LH, BA, and uterine volume. The area under the ROC curve was 0.858, supporting its utility for the initial screening of CPP in girls. However, due to the adjustment of diagnostic criteria for CPP in Chinese girls, the applicability of the previous prediction model is tentative. Insofar as previous studies did not provide specific means of using past models, this study developed novel diagnostic prediction models for CPP diagnosis using machine learning algorithms based on multi-center data, while aiming to retain the minimal number of essential predictors.

## Materials and methods

### Participants

This was a retrospective observational study. The medical records of girls with PP from January 2014 to June 2023 in Fuzhou First General Hospital Affiliated with Fujian Medical University (Fuzhou Children’s Hospital of Fujian Medical University) (Center 1), Shenzhen Children’s Hospital (Center 2), The First Affiliated Hospital, Sun Yat-sen University (Center 3), and Henan Children’s Hospital Zhengzhou Children’s Hospital (Center 4) were collected. The four hospitals were all tertiary comprehensive hospitals, and the patients were drawn from surrounding provinces and cities. All participants were diagnosed as PP by the National Consensus Statement in China (3). Breast development occurred before the age of 7.5 years. Gonadotropin-releasing hormone (GnRH) stimulation test was performed using gonadorelin with the dosage of 2.5 μg per kilogram, and a maximum limit of 100 μg. The exclusion criteria were: (1) traumatic injuries. (2) severe organs dysfunctions or any non-hormone disorder potentially affecting growth. (3) incomplete data.

The participants from Center 1 were randomly divided into two groups: training group (80%), and validation group (20%). The participants enrolled from Center 2, Center 3, and Center 4 were assigned to test group 1, test group 2, and test group 3, respectively. The training group was used for model development, the validation group was used for model parameter adjustment as well as internal validation, and the test groups were used for external validation of the models.

### Medical records

The clinical data at the first visit before treatment was recorded, including: (1) basic information: chronological age (CA), disease course. (2) physical examination: height, weight, Tanner breast stage. (3) serum hormones: basal LH and FSH, peak LH and FSH. (4) imaging findings: BA, uterine volume and ovarian volume. In addition, height standard deviation score (SDS), peak LH/FSH, and height SDS for BA (HtSDS_BA) were calculated. BA and predicted adult height (PAH) were estimated by two experienced pediatric endocrinologists using the Tanner-Whitehouse 3 (TW3) method. Uterine volume and ovarian volume were measured by a single experienced sonographer by color ultrasound using the ellipsoid formula: volume = width * length * thickness * 0.5233.

### Data analysis

Data are expressed as the mean ± SD. Differences between groups were analyzed using the Mann-Whitney U test, Fisher’s exact test, and Chi-square. A value of *P* < 0.05 represents a statistical difference. Statistics were performed using SPSS 25.0, R 4.3.0 and Python 3.8. The study is in concordance with TRIPOD+AI statement for clinical prediction models (12).

Model development involved the following steps: (1) Candidate predictors transformation: The candidate predictors were transferred mathematically according to the distribution of data. (2) Predictors screening: Lasso regression analysis was performed to screen predictors. (3) Model fit: The fitting of the models was performed with the predictors from the screening step and methods of logistic regression, linear support vector machine (SVM), RandomForest, XGBoost and fully connected neural network. The parameters of fully connected neural network model were adjusted based on the data from validation group. Model fitting was performed using Python’s scikit-learn library. For the logistic regression model, the liblinear solver was employed with L2 regularization to mitigate overfitting. The SVM utilized a Gaussian radial basis function kernel, while the RandomForest algorithm was configured with 300 decision trees and a maximum of 8 features per split. The XGBoost model was trained with 50 decision trees at a learning rate of 0.1. Additionally, The fully connected neural network model was implemented with two hidden layers containing 8 and 4 neurons respectively, using the SeLU activation function for hidden layers and a sigmoid activation for the output layer, optimized via binary cross-entropy loss function. The output of the models included probability values and classification results. Then, permutation feature assessed the strength of these predictors. (4) Internal and external validation: Internal validation was performed using data from the validation group while external validation was performed using data from the test groups. Model discrimination was assessed with C-statistics and accuracy rates. Model calibration was assessed by calibration curves. The clinical benefit was evaluated by decision curve analysis (DCA). Also, these parameters were used to compare the degree of discrimination and calibration between models. (5) Model display: A dynamic nomogram for the best model was constructed and displayed on the website for open access based on ShinyApp (https://www.shinyapps.io/).

## Results

### Participants

The medical records of 1310 girls with PP from Center 1 were collected. The participants included 502 girls with CPP and 808 girls with non-CPP. The participants from Center 1 were randomly divided into training group (1048 cases), and validation group (262 cases). Center 2 enrolled 270 girls with PP (test group 1), comprising 127 cases of CPP and 143 of non-CPP. In the clinical diagnostic protocols of Center 3 and Center 4, GnRH stimulation tests were administered to clinically suspected CPP patients (manifesting enlarged uterine volume and advanced bone age). All or predominantly CPP girls were enrolled across both centers, with Center 3 including 278 CPP girls (test group 2) and Center 4 enrolling 290 PP girls (test group 3, including 275 CPP cases and 15 non-CPP cases).

Clinical characteristics of all participants in training group, validation group, and test groups are shown in **Table 1**. Tanner stage of breast development and pubic hair development of the five groups were recorded in **Table 2**.

**Table 1:** Clinical characteristics of all participants in this study.

Clinical characteristic	Center 1 (n = 1310)		Center 2	Center 3	Center 4
	Training (n = 1048)	Validation (n = 262)	Test 1 (n = 270)	Test 2 (n = 278)	Test 3 (n = 290)
Basic information					
CA (years)	7.26 ± 0.91	7.18 ± 0.91	7.48 ± 0.88	7.84 ± 1.01	7.17 ± 0.57
Disease course (years)	0.61 ± 0.70	0.59 ± 0.65	0.79 ± 0.62	1.07 ± 0.78	0.65 ± 0.61
Physical examination					
Height (cm)	126.60 ± 8.04	125.89 ± 8.74	130.00 ± 7.11	131.31 ± 7.99	131.89 ± 6.09
Height SDS	0.82 ± 1.29	0.75 ± 1.40	0.89 ± 0.96	0.74 ± 1.03	1.66 ± 1.07
Weight (kg)	26.28 ± 5.91	26.18 ± 6.21	28.55 ± 5.46	28.87 ± 5.74	29.62 ± 5.21
Weight SDS	0.52 ± 1.51	0.57 ± 1.59	0.81 ± 1.26	0.64 ± 1.21	0.90 ± 1.33
BMI (kg/m^2^)	16.23 ± 2.33	16.32 ± 2.43	16.77 ± 2.06	16.60 ± 2.02	16.94 ± 2.17
Hematologic examination					
Basal LH (IU/L)	0.53 ± 0.97	0.41 ± 0.67	0.91 ± 1.52	1.36 ± 1.85	1.70 ± 2.37
Basal FSH (IU/L)	2.76 ± 1.80	2.56 ± 1.69	3.99 ± 2.89	3.64 ± 2.28	3.18 ± 2.20
Peak LH (IU/L)	10.23 ± 10.71	9.41 ± 10.32	12.38 ± 13.35	21.75 ± 20.87	20.01 ± 13.97
Peak FSH (IU/L)	13.70 ± 6.11	13.13 ± 5.40	15.58 ± 6.20	12.55 ± 5.01	12.31 ± 4.34
Peak LH/FSH	0.79 ± 0.75	0.73 ± 0.68	0.80 ± 0.67	1.63 ± 1.18	1.61 ± 0.88
Imaging examination					
BA (years)	9.12 ± 1.64	9.03 ± 1.67	9.64 ± 1.22	10.27 ± 1.51	8.93 ± 1.26
BA-CA (years)	1.85 ± 1.16	1.85 ± 1.18	2.16 ± 0.88	2.43 ± 1.04	1.76 ± 1.07
HtSDS_BA	-1.37 ± 1.00	-1.42 ± 1.04	-1.28 ± 0.90	-1.61 ± 0.96	-0.23 ± 0.98
PAH (cm)	153.19 ± 5.41	152.94 ± 5.64	153.71 ± 4.88	151.92 ± 5.18	159.34 ± 5.29
Uterine volume (ml)	1.57 ± 1.21	1.49 ± 1.06	1.73 ± 1.36	3.94 ± 3.73	3.27 ± 1.56
Larger ovarian volume (ml)	2.05 ± 1.21	2.00 ± 0.92	2.54 ± 9.00	2.53 ± 1.64	2.61 ± 1.22
Diagnosis					
CPP	407	95	127	278	275
Non-CPP	641	167	143	0	15

Data are presented as mean ± SD.

**Table 2:** Sexual characteristic of all participants.

Sexual characteristic	Training (%) (n=1048)	Validation (%) (n=262)	Test 1 (%) (n=270)	Test 2 (%) (n=278)	Test 3 (%) (n=290)
Tanner breast stage					
II	771 (73.6)	190 (72.5)	166 (61.5)	43 (15.4)	160 (55.2)
III	260 (24.8)	70 (26.7)	89 (33.0)	142 (51.1)	125 (42.1)
IV	17 (1.6)	2 (0.8)	12 (4.4)	83 (29.9)	5 (1.7)
V	0 (0)	0 (0)	3 (1.1)	10 (3.6)	0 (0)
Tanner pubic hair stage					
I	1015 (96.9)	248 (94.7)	257 (95.2)	161 (57.9)	265 (91.4)
II	28 (2.7)	11 (4.2)	11 (4.1)	69 (24.8)	24 (88.3)
III	5 (0.4)	3 (1.1)	2 (0.7)	42 (15.1)	1 (0.3)
IV	0 (0)	0 (0)	0 (0)	6 (2.2)	0 (0)

### Model development

(1) Candidate predictors transformation: A total of 12 independent predictors were initially included, including CA (years), disease course (years), height SDS, BMI (kg/m^2^), Tanner breast stage, Tanner pubic hair stage, basal LH (IU/L), basal FSH (IU/L), BA-CA (years), HtSDS_BA, uterine volume (ml), and larger ovarian volume (ml). Because of skewed distribution of data, the continuous variables of disease course and basal LH were transformed into classified variables. The disease course was classified into 3 levels: “< 0.5 years”, “≥ 0.5 and < 1 years”, “≥ 1 years”, while basal LH was also classified into 3 levels: “< 0.20 IU/L”, “≥ 0.20 and < 0.83 IU/L”, “≥ 0.83 IU/L” (13). Due to the uneven Tanner breast stage and pubic hair development, these two characteristics were both regrouped. Tanner breast stage was regrouped into “B = 2” and “B > 2”, while Tanner stage of pubic hair was regrouped into “P = 1” and “P > 1”. The predictors after transformation are shown in **Table 3**.

**Table 3:** Candidate predictors after transformation.

Characteristic grouping	Training (%) (n=1048)	Validation (%) (n=262)	Test 1 (%) (n=270)	Test 2 (%) (n=278)	Test 3 (%) (n=290)
Disease course					
< 0.5 years	540 (51.5)	133 (50.8)	89 (33.0)	69 (24.8)	124 (42.8)
≥ 0.5 and < 1 years	185 (17.7)	44 (16.8)	70 (25.9)	54 (19.4)	72 (24.8)
≥ 1 years	323 (30.8)	85 (32.4)	111 (41.1)	155 (55.8)	94 (32.4)
Basal LH					
< 0.20 IU/L	548 (52.3)	143 (54.6)	68 (25.2)	60 (21.6)	37 (12.7)
≥ 0.20 and < 0.83 IU/L	337 (32.2)	83 (31.7)	117 (43.3)	97 (34.9)	131 (45.2)
≥ 0.83 IU/L	163 (15.5)	36 (13.7)	85 (31.5)	121 (43.5)	122 (42.1)
Tanner stage of breast development					
B = 2	771 (73.6)	190 (72.5)	166 (61.5)	43 (15.5)	160 (55.2)
B > 2	277 (26.4)	72 (27.5)	104 (38.5)	235 (84.5)	130 (44.8)
Tanner stage of pubic hair development					
P = 1	1015 (96.9)	248 (94.7)	257 (95.2)	161 (57.9)	265 (91.4)
P > 1	33 (3.1)	14 (5.3)	13 (4.8)	117 (42.1)	25 (8.6)

**Figure 1:**
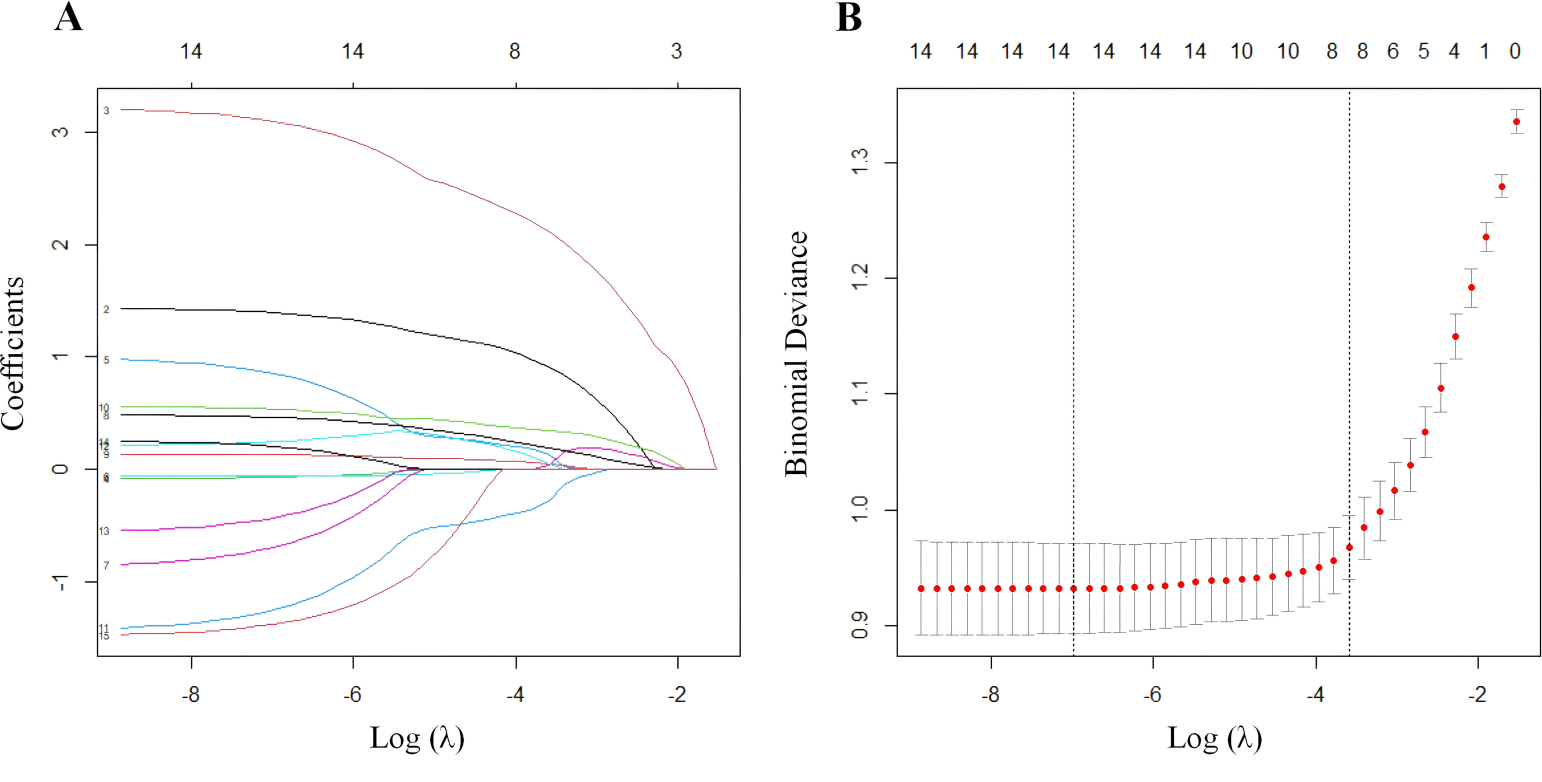
Screening of predictors based on Lasso regression. **(A)** Least absolute shrinkage and selection operator coefficient profiles of the 12 candidate predictors. **(B)** The partial likelihood deviance (binomial deviance) curve was plotted versus log (λ). The dotted lines were drawn at the optimal values by the minimum criteria and 1 standard error criteria.

**Figure 2:**
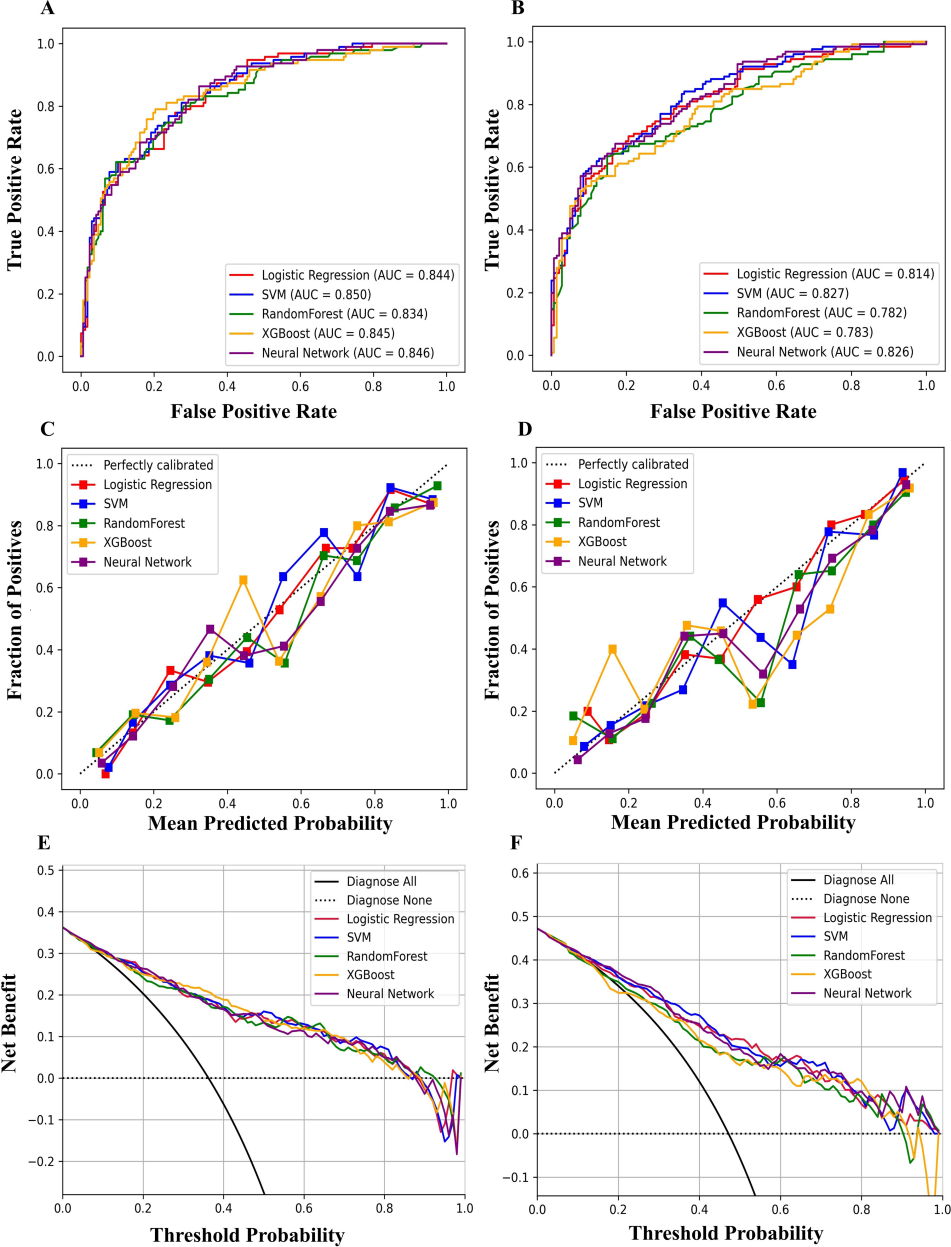
Internal validation and external validation for prediction models. **(A)** ROC curves of all prediction models in internal validation. **(B)** ROC curves of all prediction models in external validation. **(C)** Calibration curves of all prediction models in internal validation. **(D)** Calibration curves of all prediction models in external validation. **(E)** Decision curve analysis of the five prediction models in internal validation. **(F)** Decision curve analysis of the five prediction models in external validation.

(2) Predictors screening and model fit: The candidate predictors above were further screened by Lasso regression based on 1 standard error criteria of lambda (**Figure 1**). When λ = 0.00077, a total of eight independent predictors were screened, including CA, disease course, height SDS, basal LH, BA-CA, HtSDS_BA, uterine volume, and larger ovarian volume. With these predictors, models were developed with five different methods, including logistic regression, SVM, RandomForest, XGBoost and fully connected neural network. The neural network contained one input layer, two hidden layers, and one output layer. Based on relative predictability, basal LH, uterine volume, HtSDS_BA, CA, larger ovarian volume, height SDS, BA-CA, and disease course were 40.9%, 15.3%, 8.9%, 8.6%, 8.4%, 8.0%, 6.5%, 3.5% respectively.

(3) Internal and external validation: These five models were internally validated using the model validation group data to assess model performance and evaluate goodness-of-fit. Subsequently, external validation was performed using the model test groups data to compare the models’ performance and test their generalizability to external data. The ROC curves, calibration curves, and DCA curves for both internal and external validation in test group 1 are presented in **Figure 2**. the accuracy rates of these models for internal validation and external validation in 3 test groups are shown in **Table 4**.

**Table 4:** Accuracy rates of prediction models.

Models	Internal validation	External validation			
	Validation (%)	Test 1 (%)	Test 2 (%)	Test 3 (%)	Average (%)
Logistic regression	78.2	74.5	69.1	68.9	70.8
SVM	78.6	73.0	70.5	72.7	72.1
RandomForest	77.1	69.7	70.5	74.5	71.6
XGBoost	77.5	78.3	67.6	69.6	71.8
fully connected neural network	77.1	72.3	75.2	80.8	76.1

(4) Model display: The best model was replicated in the R software, and the webpage calculator was built by the “DynNom” R package on the website: https://wuwenyong.shinyapps.io/CPPpredict/.

## Discussion

The clinical diagnosis of CPP in girls requires a comprehensive assessment based on multiple determinants, including the premature appearance of secondary sexual characteristics, gonadal enlargement, serum gonadotropin and sex hormone levels within pubertal ranges, advanced BA, and accelerated linear growth. Foremost among these, the GnRH stimulation test is the preeminent diagnostic indicator for CPP. This test involves collecting a multitude of venous blood samples after GnRH administration to measure LH and FSH levels. As mentioned earlier, the GnRH stimulation test may have adverse reactions and is costly. Consequently, previous researchers have conducted a series of studies aimed at simplifying the GnRH stimulation test, or establishing ancillary diagnostic approaches, for CPP that do not rely solely on stimulation testing (13, 14). The diagnostic prediction models herein achieve of this goal.

Data from the four centers were allocated to the training group, validation group, and 3 test groups, respectively. Comparison of clinical data across the participating centers revealed that girls enrolled from Center 3 and 4 exhibited more advanced pubertal development compared to those from the other two centers. Basal LH and Peak LH levels were significantly higher in those centers. Additionally, uterine volume and Tanner stage were increased in these centers compared to the other two. This disparity is clearly attributable to differences in clinical referral pathways: Centers 3 and 4 primarily enrolled patients undergoing the GnRH stimulation test who were highly suspected of CPP. We acknowledge that this selection bias may affect the interpretation of the external validation results from these two centers. Due to obvious case enrichment, we only reported accuracy rates for Centers 3 and 4, and did not calculate prevalence-dependent metrics such as PPV. Accuracy in such highly imbalanced cohorts can be misleadingly high, as it largely reflects correct classification of CPP cases. Therefore, to evaluate model performance in a real-world balanced setting, we rely primarily on Test Group 1 from Center 2, which enrolled a nearly equal proportion of CPP and non-CPP girls. In this cohort, our models demonstrated good discrimination and calibration, supporting their generalizability.

We developed and validated new prediction models for the diagnosis of CPP girls in response to the conventional diagnostic criteria in China. Compared to the CPP diagnostic prediction model established before the revision of the diagnostic criteria and also based on the Chinese population, which included age at onset of puberty, basal LH, larger ovarian volume, and uterine volume as predictors (9), basal LH and uterine volume were the key predictors in our models. These predictors have been associated with CPP in previous studies. For example, a moderate positive correlation between basal LH and peak LH values (Spearman’s r = 0.518) were found, suggesting that basal LH may independently indicate activation of the HPGA (15). Basal LH is the foremost parameter for the diagnosis of CPP. By consensus, guideline established by multiple pediatric endocrinology societies worldwide, including China, consider a basal LH value >0.2 IU/L as a pubertal value (3, 13). Although uterine volume cannot serve as a standalone diagnostic indicator for CPP, it does bolster the diagnosis. An optimal cut-off value of uterine volume from 1 to 3 ml is consistent with CPP (3, 16, 17).

Although not as accurate as other predictors, the HtSDS_BA, CA, larger ovarian volume, and height SDS were of similar magnitude in the model, and their predictive effect in the models varied from 8.0% to 8.9%. HtSDS_BA can represent the impaired growth potential and is routinely used to predict final height and, hence, is the main indication for CPP treatment (18, 19). In girls, puberty commences between 8–12 years old with actuation of the HPGA. Ovarian enlargement is also an attendant manifestation of puberty. An ovarian volume of 1 to 3 ml is consistent with onset. While its diagnostic utility for CPP in girls is more limited than that of uterine volume, a significant correlation has nevertheless been demonstrated (3). Height SDS can partially reflex the growth rate before medical treatment. Children with CPP often have linear growth acceleration, with a resultant increase of height SDS. Furthermore, the BA-CA exceeding 1 year is commonly observed in CPP (20). Both BA-CA and disease course have been incorporated in previous prediction models (7, 10). Additionally, our model did not include advanced neuroimaging markers such as pituitary gland volume or radiomic features derived from brain MRI, which have recently been reported as potential diagnostic indicators for CPP (21, 22). We excluded these variables because, in current Chinese clinical practice, brain MRI is not routine for suspected CPP. It is performed only in selected patients with strong evidence of HPGA activation and advanced bone age. Thus, these data were largely unavailable and would have caused selection bias. Furthermore, our aim was a practical, low-cost model using widely accessible parameters. Future studies could explore whether pituitary MRI adds value to our current approach.

A total of five prediction models were developed in this study. In the internal validation of group data, the area under ROC curve of five models were from 0.850 to 0.834; these values indicate all models were sound. However, external validation demonstrated that the logistic regression, SVM, and fully connected neural network models outperformed the other two models. By comparing the accuracy rates, SVM and fully connected neural network models had superior accuracy in both internal and external validations. The calibration curves demonstrated that all models exhibited oscillations around the ideal diagonal line, indicating favorable calibration performance. DCA curves revealed comparable clinical utility across models at threshold probabilities between 0 and 0.9, with all models demonstrating favorable clinical utility. Through internal and external validations, the prediction model developed by SVM demonstrated best degree of discrimination and calibration.

This study developed pragmatic diagnostic models for CPP girls based on the diagnostic criteria in China. These models can minimizing the use of GnRH stimulation tests, thus reducing the financial burden, time involvement, and discomfort of multiple venipuncture. Compared with previous models, our models had similar performance despite modified diagnostic criteria (7, 9, 10, 23).

This study represents the largest multi-center investigation to date on predictive modeling for CPP girls, ensuring the model’s stability for widespread adaptation. However, several limitations remain. Firstly, some centers performed GnRH stimulation tests only on girls suspected of CPP, resulting in predominantly CPP cases from Center 3 and Center 4. Consequently, this subset of data could not be utilized for comprehensive model validation. Secondly, only Center 4 was located in Northern China, which may have disparate clinical evaluations, ethnologic customs, and genetic make-up than their Southern counterparts. Before clinical implementation, additional external validation from more diverse centers is warranted. Third, our model used the Chinese diagnostic threshold of 7.5 years, while many countries use 8 years. Adaptation to different thresholds requires model recalibration using local data that follow regional diagnostic criteria. International validation is needed before global implementation.

In summary, our prediction models can foretell the diagnosis of CPP in girls without GnRH stimulation test. Through the external validation of the test group data, the models showed good degree of discrimination and calibration. Future prospective clinical impact studies and expansion of sample size through broader multicenter collaborations are planned to further evaluate the model’s practical utility and generalizability.

## Consent to participate

The exemption from the informed consent requirement is permitted by the Ethics Committee of Fuzhou Children’s Hospital of Fujian Province.

## Data Availability

The raw data supporting the conclusions of this article will be made available by the authors, without undue reservation.
